# Decoding the hallmarks of allograft dysfunction with a comprehensive pan-organ transcriptomic atlas

**DOI:** 10.1038/s41591-024-03030-6

**Published:** 2024-06-18

**Authors:** Harry Robertson, Hani Jieun Kim, Jennifer Li, Nicholas Robertson, Paul Robertson, Elvira Jimenez-Vera, Farhan Ameen, Andy Tran, Katie Trinh, Philip J. O’Connell, Jean Y. H. Yang, Natasha M. Rogers, Ellis Patrick

**Affiliations:** 1 School of Mathematics and Statistics, The University of Sydney, Camperdown, New South Wales Australia; 2 Sydney Precision Data Science Centre, The University of Sydney, Camperdown, New South Wales Australia; 3 Centre for Transplant and Renal Research, Westmead Institute for Medical Research, Westmead, New South Wales Australia; 4 Charles Perkins Centre, The University of Sydney, Camperdown, New South Wales Australia; 5 Computational Systems Biology Group, Children’s Medical Research Institute, Faculty of Medicine and Health, The University of Sydney, Westmead, New South Wales Australia; 6 Kinghorn Cancer Centre and Cancer Research Theme, Garvan Institute of Medical Research, Darlinghurst, New South Wales Australia; 7 St. Vincent’s Clinical School, Faculty of Medicine, University of New South Wales, Sydney, New South Wales Australia; 8 Department of Renal and Transplantation Medicine, Westmead Hospital, Westmead, New South Wales Australia; 9 Laboratory of Data Discovery for Health Limited (D24H), Science Park, Hong Kong SAR, China; 10 Faculty of Medicine and Health, University of Sydney, Camperdown, New South Wales Australia; 11 Centre for Cancer Research, Westmead Institute for Medical Research, Westmead, New South Wales Australia

**Keywords:** Transplant immunology, Data integration

## Abstract

The pathogenesis of allograft (dys)function has been increasingly studied using ‘omics’-based technologies, but the focus on individual organs has created knowledge gaps that neither unify nor distinguish pathological mechanisms across allografts. Here we present a comprehensive study of human pan-organ allograft dysfunction, analyzing 150 datasets with more than 12,000 samples across four commonly transplanted solid organs (heart, lung, liver and kidney, *n* = 1,160, 1,241, 1,216 and 8,853 samples, respectively) that we leveraged to explore transcriptomic differences among allograft dysfunction (delayed graft function, acute rejection and fibrosis), tolerance and stable graft function. We identified genes that correlated robustly with allograft dysfunction across heart, lung, liver and kidney transplantation. Furthermore, we developed a transfer learning omics prediction framework that, by borrowing information across organs, demonstrated superior classifications compared to models trained on single organs. These findings were validated using a single-center prospective kidney transplant cohort study (a collective 329 samples across two timepoints), providing insights supporting the potential clinical utility of our approach. Our study establishes the capacity for machine learning models to learn across organs and presents a transcriptomic transplant resource that can be employed to develop pan-organ biomarkers of allograft dysfunction.

## Abstract (web summary)

A comprehensive analysis of omics data from biopsies and blood samples from more than 12,000 cases of heart, lung, liver and kidney transplants provides insights into shared mechanisms of allograft dysfunction across organs.

## Main

Organ transplantation is a crucial therapeutic option for individuals with end-stage organ failure, providing a mortality benefit and improved quality of life^1–3^. Long-term graft survival varies among organs (82% for kidney transplants^4^, 80% for liver^5^, 59% for lung^6^ and 72.5% for heart^7^), but longevity is universally limited by allograft dysfunction, a term that encompasses a broad range of pathologies. Dysfunction can be driven by ischemia reperfusion injury manifesting as delayed graft function (DGF) (or primary non-function)^8,9^, activation of the adaptive immune response, which initiates rejection and tissue destruction^10,11^, or maladaptive repair responding to injury cues that replaces functioning parenchyma with extracellular matrix and culminates in fibrosis^12,13^. Molecular hallmarks of allograft dysfunction have already been established from organ-specific human studies^14–17^, particularly kidney transplantation, which is the most frequently performed transplant surgery worldwide^18^.

Numerous technological advances have supported rapid evolution of in silico research, revolutionizing understanding of allograft pathology at a molecular level, with the promise to transform our approach to healthcare. The complex data encapsulated by high-resolution multi-omics approaches provide a global assessment of tissue microenvironments, capable of dismantling the interaction between host and recipient, and the ensuing alloimmune response^19^. Precise definitions of cell type and functional state facilitates analysis of more subtle allograft (patho)physiology compared to the limited interpretation arising from clinical and histological parameters. Despite a plethora of genomic knowledge and identification of potential biomarkers^13,20–23^, there is limited consensus among organs and restrained incorporation of these data into routine clinical practice to supersede current (non-molecular) diagnostic standards for monitoring allograft function and modifying treatment. This has unacceptable implications for transplant recipients in which their survival and/or that of the graft has not advanced substantially in the past two decades.

A critical challenge in the field lies in the assumption that transplanted organs exhibit inherent molecular heterogeneity in response to cellular injury, rejection and repair. Studies previously demonstrated that markers predictive of dysfunction in one transplant organ cohort fail to show concordance when applied to other allografts^24,25^. Analytical accuracy is further complicated by the use of different technologies to generate transcriptomic signatures^26^. To partly address these obstacles, an expansive, manually generated meta-analysis from pre-clinical and human transplant studies was performed to create the Banff Human Organ Transplant (BHOT), a gene array that reflects global allograft dysfunction^27^. However, the current lack of a definitive quantitative capacity to compare molecular associations across transplant datasets significantly hampers our ability to acquire a comprehensive understanding of clinical pathologies across all transplanted organs.

Here we introduce the concept of ‘pan-organ allograft dysfunction’, positing that pathophysiological genomic signatures are agnostic of organ type. To support this notion, we curated publicly available transcriptomic datasets across the four most common solid organs transplanted in humans, profiling three main forms of organ dysfunction (DGF, acute rejection and fibrosis), in addition to transplant tolerance, with the aim of identifying a cohort of conserved genes for each phenotype. Furthermore, we developed, implemented, evaluated and validated a novel transfer learning framework that leverages information across different organ transplants to develop a superior transcriptomic signature. We provide this comprehensive curated dataset as a publicly available resource. Combined, these resources provide an insight into the pan-organ hallmarks of allograft dysfunction.

## Results

### Pan-organ ResOurce for Molecular Allograft Dysfunction (PROMAD)

**Fig. 1:**
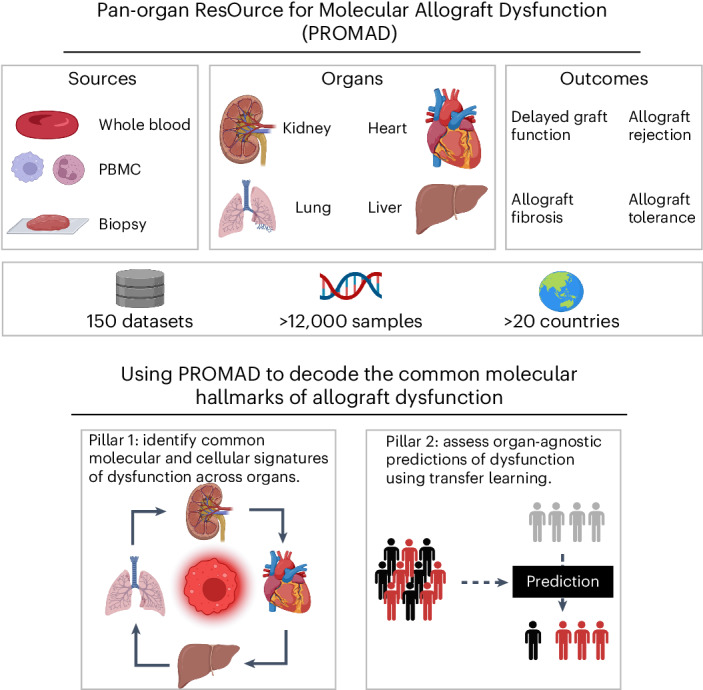
The PROMAD atlas: a comprehensive map of allograft dysfunction. The PROMAD atlas encapsulates an extensive array of data, presenting a multifaceted view of allograft dysfunction through whole blood samples, PBMCs and allograft biopsies. It comprises data from heart, lung, liver and kidney transplants, encompassing four transplant outcomes, namely DGF, rejection, fibrosis and tolerance. The collection and curation process resulted in a repository of 150 datasets consisting of 12,765 molecular samples derived from more than 20 countries worldwide. We performed analysis on PROMAD, identifying common molecular and cellular signatures of dysfunction across organs and using our novel transfer learning framework to assess the effectiveness of organ-agnostic predictions of allograft dysfunction. This figure was created with BioRender.

**Extended Data Fig. 1:**
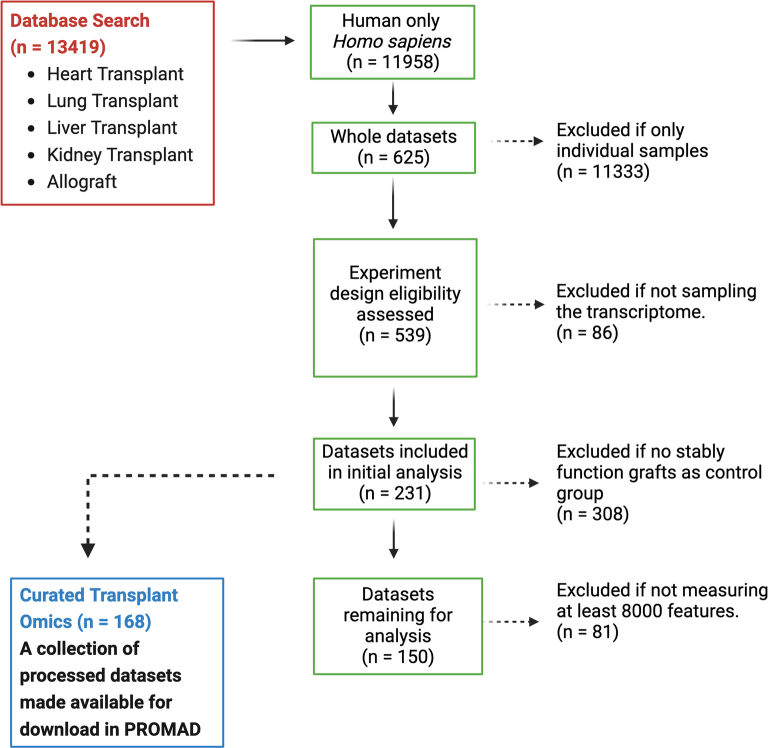
Schematic of the literature review workflow for transplant omics datasets. A systematic search of the GEO and ArrayExpress databases using terms related to heart, lung, liver, and kidney transplants, yielded 13,419 datasets. Datasets underwent scrutiny for inclusion, excluding non-human or those lacking proper controls, defined as stable functioning grafts. Data were then extracted and normalized using various methods appropriate to the data type. The PROMAD repository was created, comprising 168 processed datasets available for research access, with 150 transcriptomic datasets selected for our study. Non-coding region datasets, while excluded from this study, were also included in PROMAD.

We postulated that the pre-existing transcriptomic datasets from human samples, across multiple transplanted organs (kidney, heart, liver and lung), displaying varied clinical pathologies (DGF, acute rejection, fibrosis and tolerance) would facilitate generation of a comprehensive gene expression atlas of allograft dysfunction. We curated available datasets incorporating microarray, bulk tissue RNA sequencing (RNA-seq) and single-cell RNA-seq technologies (Extended Data Fig. 1). Our large-scale atlas comprises 150 datasets and 12,970 samples (Fig. 1 and Supplementary Table 1). This resource is publicly available via https://shiny.maths.usyd.edu.au/PROMAD/. We leveraged this atlas to identify pan-organ molecular signatures that correlate with clinically defined allograft pathologies and evaluated their effectiveness as organ-agnostic predictors of (dys)function.

### Shared molecular markers in allograft rejection

**Fig. 2:**
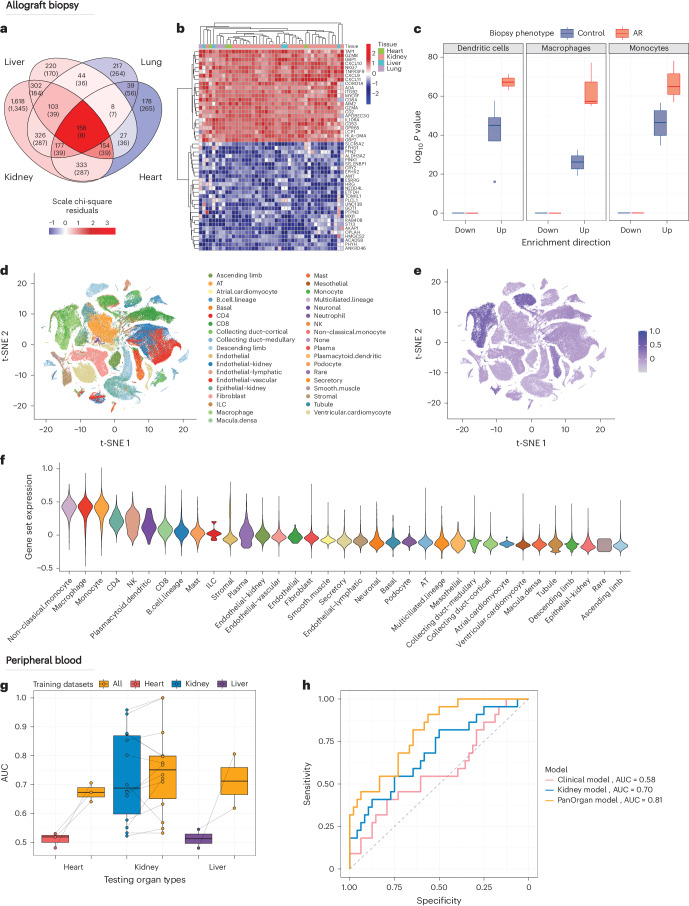
Identification of a pan-organ rejection signal across solid organ transplantation. **a**, Venn diagram showing the overlap and uniqueness of differentially expressed genes between biopsy samples from allografts experiencing acute rejection and otherwise stably functioning grafts. The number of overlapping genes (and number of genes expected by chance). **b**, Heatmap of the top 50 rejection-specific genes, with each column representing a dataset and each row a gene. **c**, Box plot of Cepo enrichment scores of genes from **b** in cell types from acute rejection and stably functioning grafts (*n* = 6 and *n* = 16 biologically independent control and allograft rejection (AR) samples were used, respectively). **d**, t-SNE plot of merged single-cell RNA-seq datasets, with cells colored by cell type classification. **e**, t-SNE plot of merged single-cell RNA-seq datasets, with cells colored by average expression of genes from **b**. **f**, Violin plot depicting the expression of rejection markers across minor cell types. The *x* axis represents different cell types, and the *y* axis represents the average expression of the rejection gene set markers from **b**. The width of each violin plot corresponds to the density of expression values for each cell type. **g**, Box plot of liquid biopsy dataset model performance measured by AUC, comparing the performance of organ-specific models from heart (*n* = 3 datasets from 65 biologically independent patient samples), kidney (*n* = 18 datasets from 2,257 biologically independent patient samples) and liver (*n* = 2 datasets from 100 biologically independent patient samples) compared to the pan-organ model (*n* = 23 datasets from 2,422 biologically independent patient samples). Each point is an evaluation of model performance on an independent dataset. Points that are joined by a line represent the same dataset. **h**, ROC plot of three models applied to AUSCAD: Pan-Organ model (trained on all peripheral blood datasets in PROMAD), Kidney-specific model (trained on all kidney transplant peripheral blood datasets in PROMAD) and Clinical model (creatinine, eGFR and serum albumin). Each model was evaluated using the AUC. Box plots in **c** and **g** show Q1, median and Q3, and the lower and upper whiskers show Q1 − 1.5× IQR and Q3 + 1.5× IQR, respectively. AT, alveolar type; ILC, innate lymphoid Cell; IQR, interquartile range; NK, natural killer; Q, quartile.

A reductionist understanding of acute rejection is that of an orchestrated adaptive immune response to the allograft, but this fails to reflect the complexity of interactions between infiltrating recipient immune cells and the donor parenchyma. Rejection is not necessarily easy to diagnose histologically due to inherent risks of tissue sampling and consensus-driven histopathological scoring systems that remain observer dependent. To detect pan-organ mechanisms of acute rejection, we used our atlas to identify consistently differentially expressed genes in allografts with biopsy-proven rejection. We identified 54 datasets encompassing 40 kidney, five lung, five liver and four heart studies, each comparing stable with acutely rejecting grafts. To avoid potential loss of biologically relevant variation unique to each study, we chose not to employ batch correction methods when combining data and, instead, employed a *P* value combination method to reduce the impact of technical artifacts between datasets^28,29^. We identified genes associated with acute rejection unique to each organ as well as a common set of 158 genes that were differentially expressed across all four organs, which was nearly 20 times higher than the eight genes expected by chance (*P* = 5.44 × 10^−8^; Fig. 2a). Genes encoding chemokines (CXCL9, CXCL10 and CXCL11), granzymes (GZMA and GZMB) and cell surface receptors (CD2, CD8A and CD53) were associated with rejection in kidney, heart, liver and lung transplants (Fig. 2b), demonstrating a unifying pan-organ molecular marker.

**Extended Data Fig. 2:**
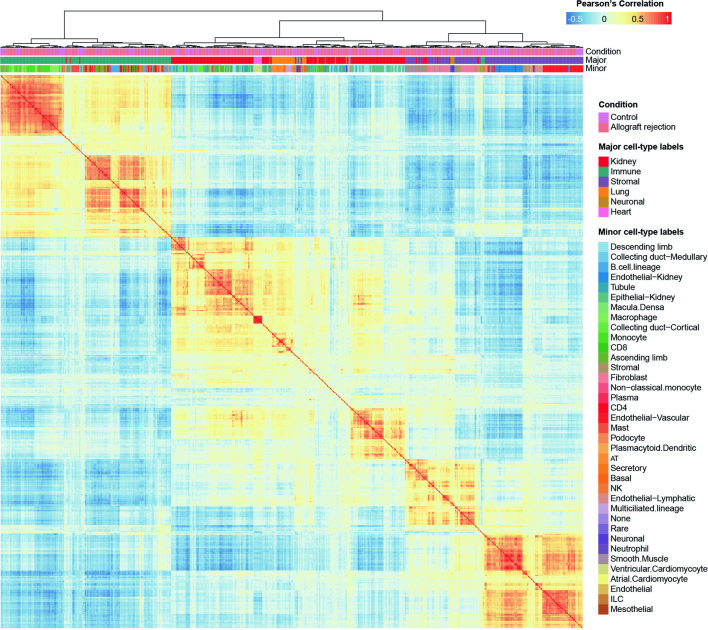
Clustering of CEPO statistics. Correlation heatmap of cell-identity gene statistics generated from Cepo (Kim et al., 2021) for each cell type across tissues and datasets in the pan-organ allograft rejection atlas. The heatmap is hierarchically clustered by the similarity of correlation profiles. Colour bars denote tissue origin or cell type of each sample.

To identify the cellular origin of this pan-organ molecular signal, we used six single-cell RNA-seq datasets across multiple solid organ transplants comparing acutely rejecting and stable allografts. The Cepo framework was used to generate cell type importance statistics for each gene in each of the 36 recognzed cell types defined in our atlas (Extended Data Fig. 2). Using Cepo statistics and our set of 158 pan-organ rejection genes, we demonstrated differential enrichment in myeloid cell subsets from biopsies with acute rejection (Fig. 2c and Supplementary Fig. 1). We then aligned cells from our PROMAD atlas to a common low-dimensional embedding (Fig. 2d and Supplementary Fig. 2) where we confirmed that the pan-organ acute rejection gene signature was highly expressed in myeloid cells (Fig. 2e,f).

### Liquid biopsy molecular markers in allograft rejection

**Extended Data Fig. 3:**
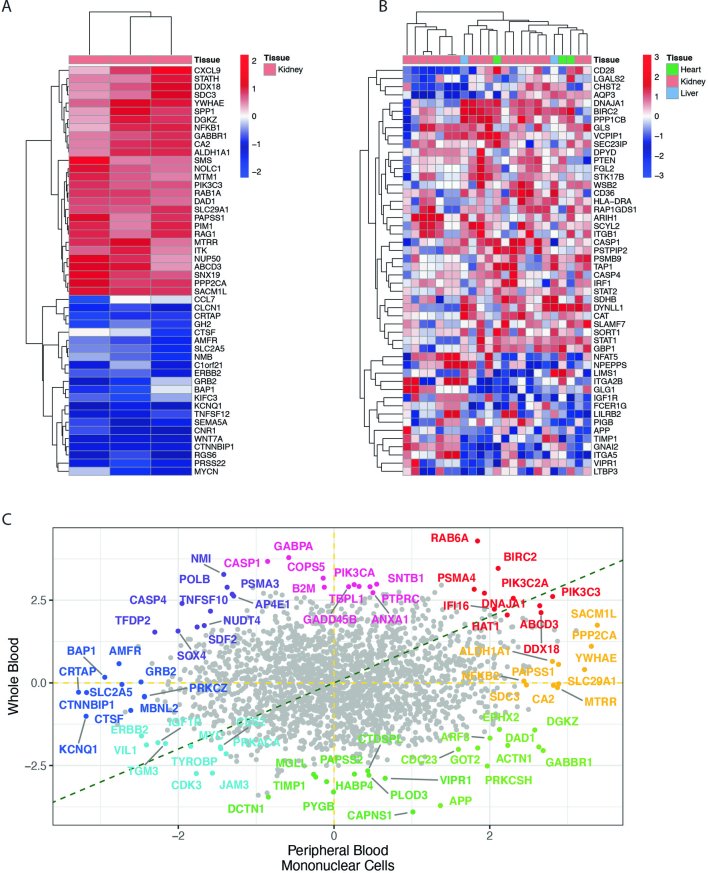
Liquid biopsy model from whole blood and PBMC in acute allograft rejection. **A**. Heatmap of top 50 genes differentially expressed in across all PBMC datasets. Each cell is coloured by normal score in each dataset. **B**. Heatmap of top 50 genes differentially expressed across all whole blood datasets. Each cell is coloured by normal score in each dataset. **C**. Scatter plot of combined association statistics for allograft rejection in whole blood and PBMC.

Minimally invasive liquid biopsy tests provide a substantial advantage for monitoring allograft health, but commercially available markers have limited sensitivity and specificity to facilitate clinical decision-making. Within PROMAD, we analyzed 23 datasets of whole blood samples, comprising two liver, three heart and 18 kidney transplants with a diagnosis of acute rejection, and we compared molecular changes to patients with stable allograft function (Supplementary Table 1) using the same approach as for tissue samples. Due to the heterogeneity of rejection phenotype classifications in datasets, across organs and over time, we deliberately classified a broad, organ-agnostic signature for rejection. We identified 77 genes that were consistently associated with an acute rejection phenotype in liquid biopsies across all organs (Extended Data Fig. 3), which was more than expected by chance (when using a combined *P* value threshold of *P* < 1 × 10^−5^), and genes were predominantly involved in inflammation (CASP1, CASP4 and IRF4) and regulation of immune function (CD28, CD36 and FCER1G) (Extended Data Fig. 3). We subsequently mapped these 77 genes onto a curated set of single-cell RNA-seq datasets derived from liquid biopsy samples within our atlas, demonstrating overexpression in CD14^+^ monocytes (Supplementary Fig. 2), in keeping with the findings in biopsy samples.

### Transfer learning identifies a pan-organ rejection model

We developed a Transferable Omics Prediction (TOP) framework to assess the reliability of predictive markers of acute rejection derived from liquid biopsies. Classification models that are constructed using reference-free methods, as opposed to traditional batch correction, have been shown to be robust to technical and biological variability^30–32^ and, thus, transferable across cohorts, biological tissues and sequencing assays^26^. Traditional batch correction methods that rely on common matrix factorization methods are not suited to building models with confounding factors (Supplementary Methods). TOP relies on a key feature engineering step that we previously showed to be self-normalizing^26^. Creation of a log-ratio matrix of the most differentially expressed genes across all datasets and leveraging these relative changes in gene expression enhance the model’s robustness. As the utility of TOP extends beyond PROMAD, we made the framework available on the Bioconductor Project^33^.

**Extended Data Fig. 4:**
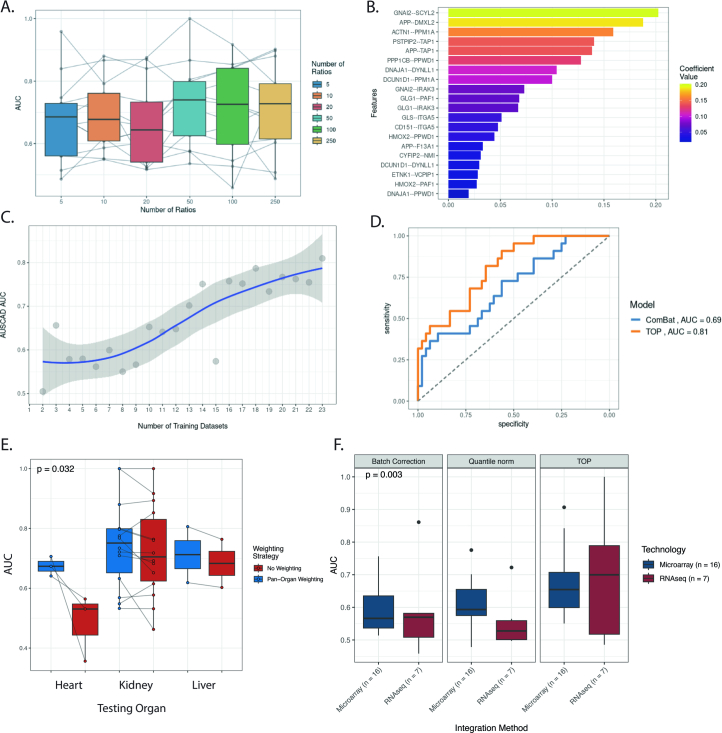
Training a liquid biopsy model using PROMAD. **A**. Boxplot of increasing number of ratios that are required to predict acute allograft rejection from liquid biopsy samples. Each point is an evaluation of model performance on an independent dataset. Points that are joined by a line represent the same dataset. Box plots show Q1, median and Q3, and the lower and upper whiskers show Q1 – 1.5 × IQR and Q3 + 1.5 × IQR, respectively. **B**. Bar plot of model coefficients for our liquid biopsy model. **C**. Dot plot of liquid biopsy model performance on the AUSCAD cohort with a loess smoothed curve representing the mean trend as the number of training datasets increases. The shaded area around the curve indicates the 95% confidence interval, reflecting the variability around the estimated mean trend. **D**. A pair of ROC curves comparing model performance on the AUSCAD cohort. Both models are trained across multiple organs, however their integration algorithms differ. TOP is coloured yellow, and ComBat is coloured blue. **E**. A boxplot of model performance across peripheral blood datasets within PROMAD (n = 23 datasets from 2422 biologically independent patient samples), when tissue weighting is applied to the TOP algorithm. Each point is an evaluation of model performance on an independent dataset. Box plots show Q1, median and Q3, and the lower and upper whiskers show Q1 – 1.5 × IQR and Q3 + 1.5 × IQR, respectively. A 3-way ANOVA using organ, dataset and weighting strategy was performed to assess if weighting strategy impacted AUC. **F**. Boxplot comparison of model performance across all datasets within PROMAD. We compare three integration algorithms (Combat, Quantile normalization and TOP). We split the model performance by technology to demonstrate TOP’s ability to cross technologies more easily. Box plots show Q1, median and Q3, and the lower and upper whiskers show Q1 – 1.5 × IQR and Q3 + 1.5 × IQR, respectively. A 2-way repeated measures anova was performed to test the impact of integration strategy on AUC.

To evaluate the impact of cross-organ learning, the TOP framework was applied to predict allograft rejection across 23 liquid biopsy datasets. Although we identified molecular mechanisms of acute rejection that are consistent across organs, it is not clear whether these markers are superior to those derived from organ-specific data. To compare their predictive performance, a pan-organ model was recursively built on 22 datasets using TOP, with one dataset being left for model evaluation. Organ-specific models were also constructed using the TOP framework and evaluated with the same leave-one-dataset-out strategy. Our findings revealed enhanced model performance for models trained on all available organs compared to solely the organs being predicted (Fig. 2g), illustrating the robustness of a pan-organ molecular signal. In conventional, organ-specific models, the mean area under the receiver operating characteristic curve (AUC) for heart, kidney and liver predictions was 0.55, 0.70 and 0.55, respectively. In contrast, the pan-organ model demonstrated improved performance (mean AUC of 0.63, 0.74 and 0.71 for heart, kidney and liver datasets, respectively). Furthermore, by varying the number of features included in the models, only 50 gene ratios were required to construct effective models (Extended Data Fig. 4). These results demonstrate the potential of cross-organ learning as a valuable approach to improved accuracy and applicability of models predicting allograft rejection.

**Extended Data Fig. 5:**
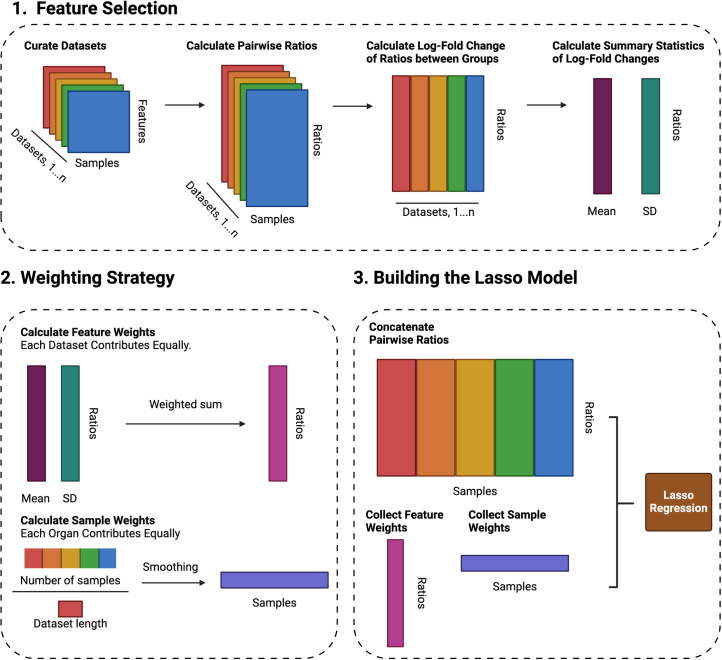
**Workflow of the Transferable Omics Prediction (TOP) framework utilised to build a regression model across multiple datasets**.

To determine the impact of dominance of kidney allograft data in PROMAD on a pan-organ biomarker, we performed a comparative analysis of weighting schemes. We employed multiple weighting strategies to ensure equal contribution from the training sets of each organ (Extended Data Fig. 5). The benefits of equal organ weighting became evident when contrasted against a naive integration strategy, which resulted in a kidney-dominant model due to the distribution of the training set. Notably, performance in kidney datasets was superior when other organs were weighted (Extended Data Fig. 4), further showcasing the advantages of adopting an organ-agnostic diagnostic approach over organ-specific models.

The benefits of a reference-free data integration method, such as the TOP framework, became apparent when considering data integration across platforms (microarray and RNA-seq). To demonstrate how TOP allows for integration across platforms, we compared other integration methods with our TOP-based approach. We demonstrate that TOP, with its ratio-based normalization, facilitated cross-technological application more adeptly than naive normalization and batch correction methods (Extended Data Fig. 4).

### Validation of a pan-organ liquid biopsy for allograft rejection

Current biomarkers in organ transplantation are limited in their ability to inform clinical decision-making^34^; however, high-throughput assays offer a potential method for biomarker identification. We validated our pan-organ findings using the Australian Chronic Allograft Dysfunction (AUSCAD) study, a prospective, single-center study of kidney and kidney–pancreas transplant recipients. This cohort contains clinical and histopathological data as well as paired 3-month protocol biopsies and blood collected and sequenced from *n* = 70 patients.

We compared the performance of three models in predicting acute rejection in AUSCAD: a logistic regression model built on clinical data (estimated glomerular filtration rate (eGFR), creatinine and serum albumin), a TOP model (trained on PROMAD data from kidney transplant patients) and, finally, our TOP model trained on all samples (Pan-Organ liquid biopsy model). We observed that our pan-organ model (AUC = 0.81) outperformed both gold-standard clinical information (AUC = 0.58) and kidney-specific models (AUC = 0.70) in predicting rejection from whole blood samples (Fig. 2h). These results underscore the diagnostic capability of a pan-organ model, positioning it as a potential alternative to both traditional and organ-specific methodologies. However, our intention is not to provide an alternative to a biopsy but, rather, to demonstrate the benefit of adopting a non-invasive prediction tool that leverages information across organs.

### Molecular characteristics of allograft fibrosis

**Fig. 3:**
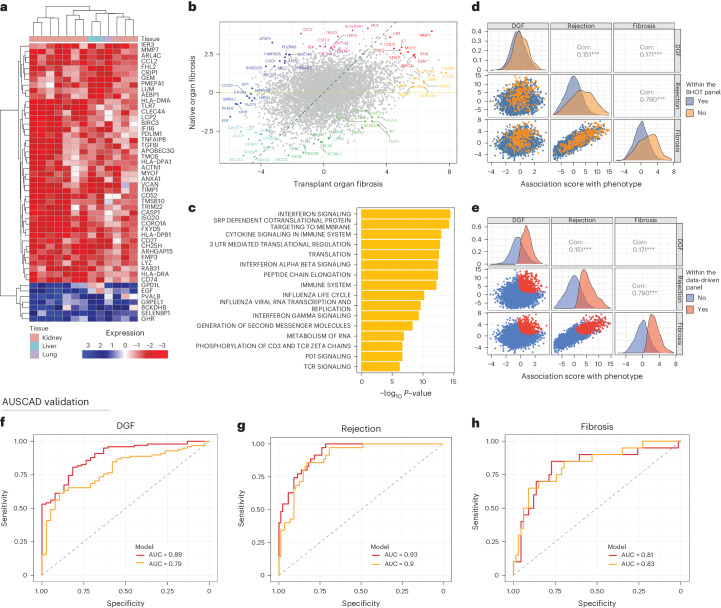
PROMAD identifies a global indicator of dysfunction in allografts. **a**, Heatmap of the top 50 fibrosis-specific genes, with each column representing a dataset and each row a gene. **b**, Scatter plot of association statistics between native and transplant organ fibrosis. The top 10 genes in each direction, indicating their degree of change between fibrotic and stably functioning grafts, are highlighted. **c**, Bar plot of pathways enriched for genes that are differentially expressed in transplant organ fibrosis but not in native organ fibrosis. Gene set enrichment was evaluated using a two-sided Wilcoxon rank-sum test. Each bar represents one Gene Ontology pathway where *P* values were adjusted for multiple comparisons using Benjamini–Hochberg correction. **d**,**e**, Pair plots of genes associated with DGF, acute rejection and fibrosis when compared to stable functioning grafts. The points in **d** are colored according to their appearance in the BHOT NanoString panel (orange), and genes in **e** are red if they appeared in the data-derived gene set. The top right panels show the correlation (Corr.) of association statistics for each gene. ROC curves compare BHOT (orange) and the data-derived panel (red) in predicting DGF (**f**), biopsy-proven acute rejection (**g**) and biopsy-proven fibrosis using the AUSCAD study as an external validation cohort (**h**). ROC, receiver operating characteristic.

Fibrosis is a maladaptive repair process occurring in response to tissue injury, characterized by excessive deposition of extracellular matrix that significantly challenges the long-term success of organ transplantation. To investigate the molecular characteristics of pan-organ allograft fibrosis and to determine whether its genomic signature differed from native organ fibrosis, we curated 14 datasets from liver, kidney and lung allografts with biopsy-proven fibrosis compared to stable graft function (Supplementary Table 1). We identified 57 genes that were differentially expressed across all organs (when using a combined *P* value threshold of *P* < 1 × 10^−7^), with increases in inflammation (CASP1, TLR7 and TNFAIP8), cell surface markers involved in immune recognition (CD27, CD52 and CD74) and HLA (Fig. 3a). These findings support the notion that immune cell activity is a significant contributor to the development of allograft fibrosis.

**Extended Data Fig. 6:**
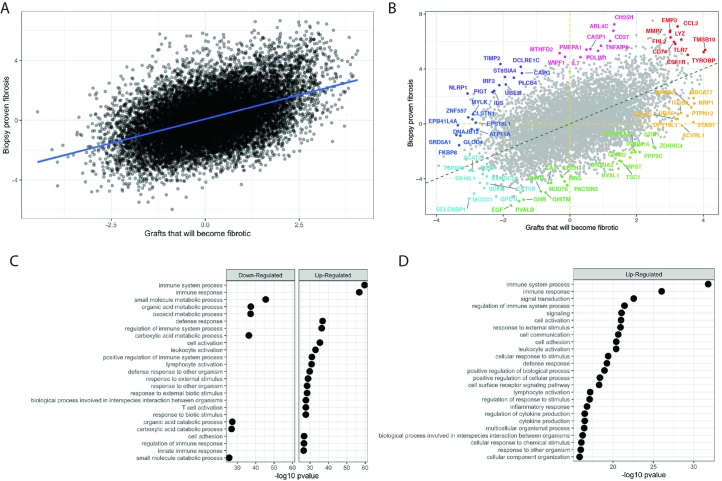
Concordance between expression profiles of biopsies that are fibrotic, compared with grafts that will become fibrotic. **A**. Scatter plot of association statistics between grafts that are fibrotic and grafts that will become fibrotic. Each point is a gene, where the R2 = 0.21, p < 0.0001. **B**. Scatter plot of association statistics between grafts that are fibrotic and grafts that will become fibrotic. A hypothesis test is performed in all 8 directions, and the top 10 genes in each direction are coloured. **C**. Dot plot of a two-sided Wilcoxon-rank sum test for pathways associated with biopsy proven fibrosis compared to stable functioning grafts. Each dot represents one reactome pathway where p-values were adjusted for multiple comparisons using the benjamini-hochberg correction. **D**. Dot plot of a two-sided Wilcoxon-rank sum test for pathways associated with stable functioning biopsies that became fibrotic compared with biopsies that remained stable. Each dot represents one reactome pathway where p-values were adjusted for multiple comparisons using the benjamini-hochberg correction.

It is unclear whether similar immunological activity portends development of fibrosis, and we investigated whether our gene set was predictive of fibrosis in stable allografts. Our atlas contained three datasets where grafts were prospectively followed for development of fibrosis. There was high concordance between genes that were predictive of fibrosis and those differentially expressed in fibrotic grafts (Extended Data Fig. 6), demonstrating a conserved process that underpins chronic allograft injury. We then explored whether this signature was preserved in native organs (Supplementary Table 2). Although the analysis demonstrated some conserved genes (Fig. 3b), there were significant disparities in allograft fibrosis with expression of immune-related pathways, including interferon signaling and T cell receptor activation (Fig. 3c).

**Extended Data Fig. 7:**
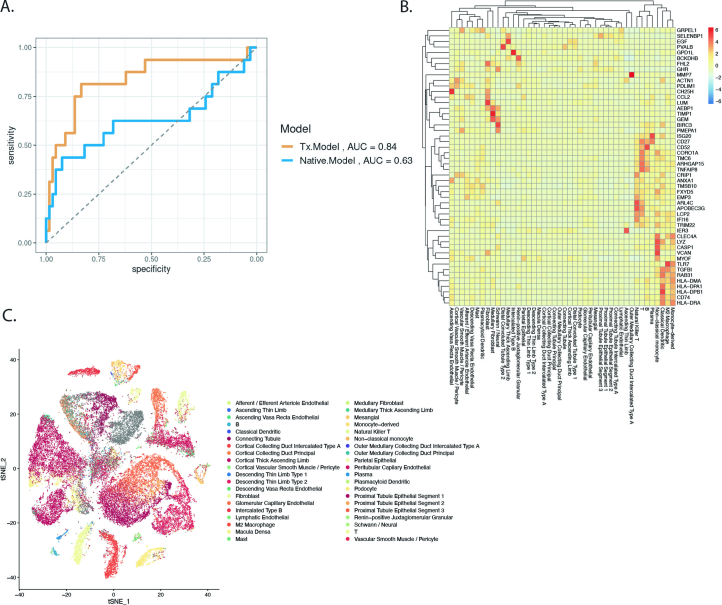
Pan-Organ fibrosis gene set at single cell resolution. **A**. ROC plot predicting biopsy proven fibrosis (IFTA > 10%) in protocol biopsies from the AUSCAD cohort. Yellow = model trained on transplant fibrosis dataset (n = 14) from the PROMAD atlas. Blue = model trained on transplant fibrosis dataset (n = 3) from the PROMAD atlas. **B**. Heatmap of fibrosis related gene (from Fig. 3A) expression in minor cell types of the pan-organ allograft dysfunction atlas **C**. tSNE projection of the cells of the pan-organ allograft dysfunction atlas. Single cells are coloured by minor cell types, as defined by our PROMAD atlas.

We then used single-cell RNA-seq data from fibrotic kidneys within PROMAD and compared our pan-organ fibrosis markers (Fig. 3a) against the expression profiles within kidney cell types (Extended Data Fig. 7). T cells and macrophages demonstrated enriched expression of fibrosis-associated genes (Extended Data Fig. 7), supporting the hypothesis that immunological activation drives allograft fibrosis and representing a potential therapeutic niche.

### Comprehensive pan-organ dysfunction gene set

**Extended Data Fig. 8:**
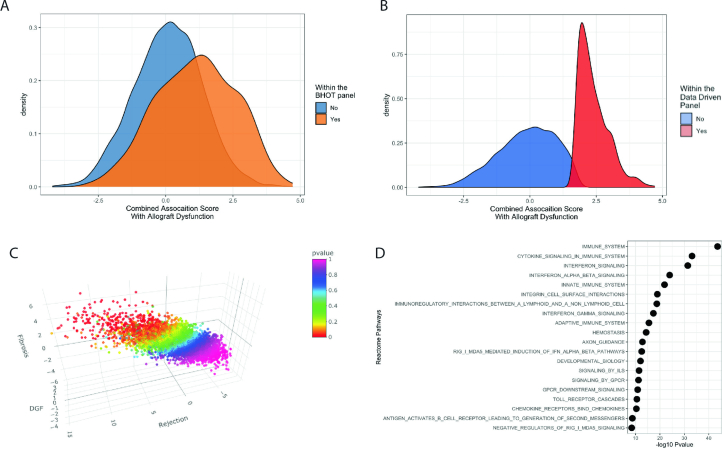
Quantitative analysis reveals a set of genes associated with Global Indicators of Dysfunction in Allografts. **A**. Density plot of association statistics for global allograft dysfunction, and genes within the Banff Human Organ Transplant (BHOT) Nanostring panel are coloured in orange. **B**. Density plot of association statistics for global allograft dysfunction, and genes within the data-driven gene set panel are coloured in red. **C**. A 3D scatter plot of association statistics for delayed graft function (DGF), allograft rejection and fibrosis. Each point is a gene, coloured by p-value for its significance to be upregulated in each condition. **D**. Dot plot of a two-sided Wilcoxon-rank sum test for our data-driven gene set using the reactome database. Each dot represents one reactome pathway where p-values were adjusted for multiple comparisons using the benjamini-hochberg correction.

Using PROMAD, we evaluated the performance of an established diagnostic tool, the BHOT panel, and compared the performance of a data-derived alternative. The BHOT panel is a manually curated array of 770 genes generated to identify allograft injury. To compare BHOT’s robustness in diagnosing general allograft injury, we first ranked each gene in order of combined change across three allograft pathologies in PROMAD (DGF, acute rejection and fibrosis). This analysis demonstrated substantial concordance between gene expression from acutely rejecting and fibrotic grafts (Extended Data Fig. 8), and the BHOT panel was able to clearly separate these pathologies (Fig. 3d). However, there was limited capacity to differentiate DGF. Acknowledging this limitation, we constructed a data-derived panel that surveyed global allograft dysfunction. We identified a set of 500 genes that were overexpressed in these pathologies across all organ transplants. This new data-derived gene set contains 400 genes not currently used in the BHOT diagnostic panel (Extended Data Fig. 8) that was able to identify changes in all selected forms of allograft dysfunction (Fig. 3e).

### Validating the data-derived gene set using the AUSCAD cohort

Using prospectively collected kidney allograft biopsy samples from AUSCAD, we compared our data-driven gene set from PROMAD with the established BHOT genes in delineating DGF, acute rejection and fibrosis. The data-driven gene set was able to predict DGF in this validation dataset (AUC = 0.89; Fig. 3f) compared to BHOT (AUC = 0.79; Fig. 3f). Furthermore, BHOT and the data-driven gene set performed equally well in classifying acute allograft rejection (AUC = 0.90 versus AUC = 0.93) (Fig. 3g) and fibrosis (AUC = 0.83 versus AUC = 0.81) (Fig. 3h).

### Biomarkers of allograft tolerance and viability

**Fig. 4:**
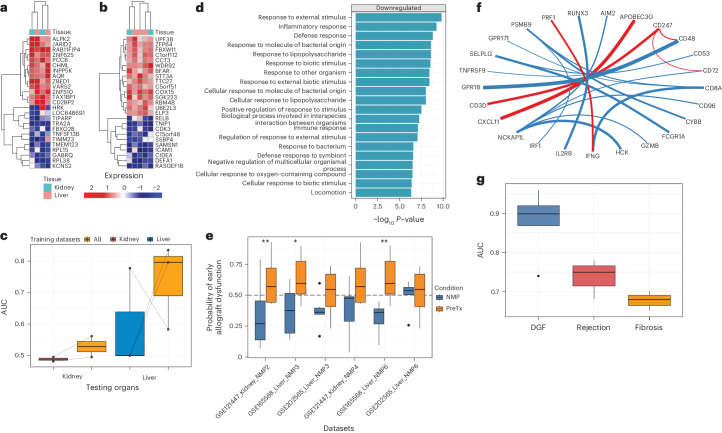
The PROMAD atlas reveals pan-organ markers for allograft tolerance. **a**,**b**, Heatmaps of the top 20 genes implicated in allograft tolerance, with each column representing a dataset and each row a gene. **a** corresponds to datasets that sampled PBMCs, and **b** corresponds to whole blood datasets. **c**, Box plot of model performance measured by AUC, comparing the performance of organ-specific kidney (*n* = 2 datasets from 68 biologically independent patient samples) and liver (*n* = 3 datasets from 52 biologically independent patient samples) models compared to the pan-organ model (*n* = 5 datasets from 120 biologically independent liver and kidney patient samples). Each point is evaluation of model performance on an independent dataset. Points that are joined by a line represent the same dataset. **d**, Bar plot of pathways that are enriched for genes differentially expressed in whole blood from tolerant recipients. Gene set enrichment was evaluated using a two-sided Wilcoxon rank-sum test. Each bar represents one Gene Ontology pathway where *P* values were adjusted for multiple comparisons using Benjamini–Hochberg correction. **e**, Box plot of predicted early allograft dysfunction risk on a logit scale. Each dataset contained biopsy samples before and after NMP. A two-sided *t*-test was used to determined significance levels between the groups (***P < 0.001, **P < 0.01 and *P < 0.05). Datasets had a varying number of biologically independent samples before and after NMP, respectively (*n* = 10, 10, *P* = 0.006; *n* = 6, 6, *P* = 0.041; *n* = 5, 10, *P* = 0.114; *n* = 6, 6, *P* = 0.157; *n* = 6, 6, *P* = 0.008; *n* = 5, 6, *P* = 0.793). **f**, Network plot of model coefficients for predicting DGF. Each line joins the two genes into a ratio, where the weight of the line corresponds to the magnitude of the model coefficients. Lines in red and blue are positive and negative coefficients, respectively. **g**, Box plot of model performance (AUC) from pre-transplant biopsies in predicting DGF (*n* = 7 datasets from 279 biologically independent patient samples), acute rejection (*n* = 3 datasets from 195 biologically independent patient samples) and fibrosis (*n* = 2 datasets from 124 biologically independent patient samples). Box plots from **c**, **e** and **g** show Q1, median and Q3, and the lower and upper whiskers show Q1 − 1.5× IQR and Q3 + 1.5× IQR, respectively. IQR, interquartile range; PreTx, pretransplantation; Q, quartile.

Our curated atlas included eight datasets from spontaneously tolerant transplant recipients (five datasets from whole blood and three datasets from peripheral blood mononuclear cells (PBMCs)) (Supplementary Table 1). True biological tolerance in organ transplantation occurs infrequently (with the exception of liver allografts)^35–38^. Recognizing its rarity, we employed PROMAD to explore the potential benefits of pooling datasets from this uncommon outcome. We identified 38 genes that were differentially expressed across whole blood (Fig. 4a) and 45 genes that were expressed across the remaining three PBMC datasets (Fig. 4b). Both gene signatures implicated suppression of the immune response and regulation of T cell proliferation (Fig. 4d) common to both kidney and liver transplant tolerance. Building on our previous observation that transfer learning models constructed from peripheral blood were capable of leveraging information across organs, we assessed this capacity in the context of allograft tolerance. Our findings revealed enhanced model performance when trained on all available organs compared to only the organ being predicted (Fig. 4c), again underscoring the benefits of a pan-organ framework in identifying allograft outcomes.

### Predicting long-term graft outcomes from implantation data

Pre-implantation biopsies, particularly for donor kidneys, have been used to determine organ quality, particularly in the context of marginal donors. Several studies correlated baseline histological characteristics with post-transplant function^39,40^ and graft survival^41,42^. We constructed a TOP model from seven datasets comprising 279 pre-implantation biopsies from liver and kidney transplants (Supplementary Table 1). Each dataset compared pre-implantation molecular markers from grafts with DGF to grafts that functioned immediately. Genes most predictive of DGF included immune cell surface markers (CD3D, CD48, CD52 and CD72) (Fig. 4f). DGF can be mitigated through the use of machine perfusion technology that provides metabolic support for the allograft. We then employed the pan-organ prediction model to calculate the probability of an organ with a pre-implantation biopsy developing DGF before and after normothermic machine perfusion (NMP). Notably, the probability of DGF developing decreased significantly after brief (<2 h) versus longer (>6 h) periods of NMP (Fig. 4e). Our model was also effective in predicting DGF or primary non-function in liver and kidney transplants (AUC = 0.89) (Fig. 4g).

## Discussion

In this study, we provide a comprehensive integration of transcriptomic data from multiple solid organ transplants, demonstrating the potential of a consolidated resource. By assembling an unprecedented 150 transcriptomic datasets, encompassing more than 12,000 samples, across the four most commonly transplanted organs in humans, we successfully identified shared molecular signatures relevant to allograft rejection, fibrosis, DGF and tolerance. In addition to these findings, we developed a novel transfer learning framework capable of borrowing information across organs that provides a harmonized coordinated analysis. Using this framework, we demonstrate the potential of pan-organ molecular signatures that can subsequently be interrogated in pre-clinical studies and adapted for clinical use as biomarkers. The signatures consistently outperformed organ-specific models and pre-existing gene panels that are transitioning from theoretical to commercial use, attesting to the translational potential of a pan-organ paradigm. These analytical vignettes also illustrate the utility of a comprehensive pan-organ atlas to validate, identify or develop transcriptomic signatures that align with known allograft pathologies.

Despite considerable advances in technologies that provide detailed molecular information, the field has failed to methodologically leverage these data in a way that improves diagnostic accuracy and guides clinical decision-making. Interrogation of individual organs analyzed by different technologies, such as NanoString, microarray or RNA-seq, has hindered identification of unified pathophysiological processes that contribute to allograft dysfunction and failure. Identification of conserved pathways that govern the interactions between donor parenchyma and the recipient immune response will influence cellular and molecular understanding, enable application to clinical practice and design of surrogate endpoints for clinical trials as well as expedite the development of therapeutic and biomarker opportunities.

A pivotal finding in our study, made possible through the interrogation of PROMAD, is the identification of a common myeloid cell population as the origin of our pan-organ molecular markers relevant to acute rejection. These data support previous publications in pre-clinical transplant models demonstrating that CD8^+^ effector T cell migration in the context of bone-marrow-derived antigen presenting cell (APC) presentation of alloantigen^43^ is necessary for monocyte-derived APC maturation^44^ and initiation of rejection^45^, and myeloid cells acquiring immunologic memory are a barrier to transplantation success^46^. The breadth of data offered by PROMAD has allowed us to translate and validate these findings to human allografts, which has been corroborated in previous studies demonstrating APC–T cell co-localization in kidney transplants^47^, increased prevalence of CD16^+^ monocyte/macrophages in rejecting heart transplants^48,49^ and resident macrophages tailoring immune responses to lung allografts^50^. Furthermore, our liquid biopsy sample analysis revealed concordant transcriptomic changes in myeloid cells. Although the differentially expressed genes between blood and tissue samples were distinct, our analysis of the PROMAD atlas has revealed therapeutically actionable insights, emphasizing that targeting myeloid-specific responses presents a viable alternative to modulate alloimmunity^51,52^.

The identification of consistent molecular markers of fibrosis across solid organ transplants, and separation of transcriptomic differences between native organ and allograft fibrosis, implicate persistent immune activation as a primary pathological driver of chronic transplant dysfunction. Inflammation, regardless of the inciting event (ischemia reperfusion injury, acute rejection) is a consistent feature of functional decline in renal^11,53–55^, lung^56,57^ and heart^58^ allografts. These findings serve as a basis for further research into molecular drivers of allograft fibrosis and the potential for limiting disease progression through targeted immunosuppression. Our study also demonstrates the potential of the TOP model to predict longer-term graft dysfunction from initial biopsy samples, including the role of therapeutics (for example, NMP) in improving graft outcomes^56,57^ and heart allografts^58^. These findings serve as a basis for further research into molecular drivers of allograft fibrosis and the potential for limiting disease progression through targeted immunosuppression.

Our study has several limitations arising from our analysis of PROMAD as a curated atlas of publicly available data. PROMAD provides evidence of shared molecular signatures of dysfunction across organs; however, these findings have not yet been explored in complementary experimental work. Although we established the effectiveness of pan-organ signatures to predict multiple allograft pathologies using a leave-one-dataset-out cross-validation (LOOCV) strategy, and further validation using AUSCAD, confirmation of our findings in prospectively recruited cohorts across other transplanted organs would increase confidence in their reliability. Moreover, the lack of detailed phenotypic data made publicly available, such as specific immunosuppression regimens or comprehensive donor histories, limits our ability to fully account for these variables in the current study. Another limitation is the detail of sample annotations within PROMAD, which is restricted to what has been publicly shared, which is particularly relevant to a diagnosis of rejection. However, we anticipate that the sample annotations made available will enable researchers to further explore the molecular understanding of different rejection phenotypes, which have distinct clinical implications^59,60^.

Variations in pathology classification have led to misinterpretation of biomarker performance on isolated external validation datasets^24,26^, a common issue faced by the transplant research community. To tackle this challenge, we developed an interactive web platform for PROMAD that enables users to assess the performance of proposed models across a comprehensively curated dataset before they progress evaluation in prospectively recruited cohorts. Our atlas provides a resource that can standardize the performance evaluation of diagnostic tools for allograft dysfunction. Finally, regardless of re-processing of all datasets through standardized pipelines, we have not performed cross-dataset normalization, instead opting for transfer learning approaches to analyze across datasets. A fully curated PROMAD atlas now provides additional opportunities to perform more sophisticated normalizations or project datasets onto common embeddings that may uncover more complex transcriptomic associations.

PROMAD provides a valuable resource for the transplant research community, compiling 150 datasets and 12,765 sequencing samples across the four most commonly transplanted organs, and the capacity to explore the landscape of allograft dysfunction. This study advances understanding of allograft dysfunction by demonstrating conserved molecular signatures across organs. PROMAD provides a resource for robust validation of prospective biomarkers as well as development of more effective diagnostic tools, risk stratification parameters and therapeutic targets.

## Methods

Our research complied with all relevant ethics regulations. The AUSCAD research protocol subject to ethics approval was approved by the Western Sydney Local Health District Human Research Ethics Committee (HREC/12/WMEAD/190).

### Data curation and creation of the PROMAD

A search to identify publicly available gene expression data in the Gene Expression Omnibus (GEO) (https://www.ncbi.nlm.nih.gov/geo/) and ArrayExpress (https://www.ebi.ac.uk/arrayexpress/) was performed using the following terms: ‘kidney transplant’, ‘liver transplant’, ‘lung transplant’, ‘heart transplant’ and ‘allograft’. Microarray, RNA-seq and single-cell RNA-seq technologies were included, revealing a total of 231 datasets published before September 2022. These datasets, all derived from human studies, were filtered based on sample size, quality and availability of clinical metadata. Detailed inclusion and exclusion criteria can be found in Extended Data Fig. 1. Upon evaluation, 150 datasets met the criteria and were incorporated into the PROMAD atlas (Supplementary Table 1).

### Statistics and reproducibility

#### Analyzing microarray datasets

We obtained the intensity-level data for each included dataset from the GEO or ArrayExpress. A log_2_ transformation was used to scale the data, and quantile normalization was used to normalize sample-specific technical artifacts. Differential gene expression within each dataset was quantified. Moderated test statistics were calculated using the eBayes function from the limma package^61^. The Benjamini–Hochberg procedure was used to control for the false discovery rate at a 5% level.

#### Analysing RNA-seq datasets

Unnormalized count datasets were obtained from the GEO or ArrayExpress. Genes with no expression in any sample were filtered from the dataset. The trimmed mean of m-values (TMM)^62^ was used to normalize library sizes of each sample. Within each dataset, differential gene expression analysis was performed using limma-voom^63^ to calculate moderated test statistics for each gene within each dataset. The Benjamini–Hochberg procedure was used to control for the false discovery rate at a 5% level.

#### Analyzing single-cell RNA-seq datasets

Unnormalized count data were obtained from the GEO or ArrayExpress and were scaled using a log transformation. Cells with no expression across all genes were filtered, and the remaining cells were annotated using Seurat. In brief, Seurat uses the Azimuth database to annotate each dataset with cell types using a dataset appropriate to the organ of interest^64^. For cells annotated as a T cell, we performed subannotation to distinguish CD4^+^ and CD8^+^ T cells. In brief, we subsetted the integrated atlas for only T cells. Using the top 2,000 variable features, we performed principal component analysis (PCA) reduction and Louvain clustering (resolution 0.05) on the *k*-nearest neighbor graphs (*k* = 20) generated from the first five principal components (PCs). On the basis of the expression of markers, the Louvain clusters were then classified into either CD4 or CD8 T cells.

#### Merging single-cell RNA-seq datasets

To embed the single-cell transcriptomes into a shared latent space, for each batch the count matrix was first normalized by the total number of reads and then multiplied by a 10,000scaling factor. The top 2,000 features were prioritized by their variance across all the single-cell RNA-seq batches. The cell pairwise anchor correspondences between different single-cell transcriptome batches were identified with 30-dimensional spaces from reciprocal PCA^65^. Using these anchors, the single-cell RNA-seq datasets were integrated and transformed into a shared space. Gene expression values were scaled for each gene across all integrated cells and used for PCA. For the integration of the organ datasets, k.anchor, k.filter and k.weight were set to 5, 200 and 100, respectively. After merging all datasets, we performed a t-distributed stochastic neighbor embedding (t-SNE) dimension reduction.

#### Identifying common differentially expressed genes in datasets

To combine the moderated test statistics of each gene, across all datasets, we used the directPA package^28^. In brief, a normal transformation was applied to the test statistics for each dataset, converting limma test statistics to normal z-scores. Stouffer’s method was used to combine the z-scores across all datasets for each gene.

#### Calculating expected intersection of differentially expressed genes

To calculate the expected number of overlapping differentially expressed genes, we first calculated the marginal probabilities of a gene being differentially expressed in each organ. The product of these marginal probabilities was then used to determine the expected number of genes that should be common among organs, by chance alone. A chi-squared test was used to determine if the number of observed overlapping genes was different than what was observed.

#### Pathway analysis

In the process of aggregating a set of genes across datasets (whether common or unique), the directPA pipeline returned a combined z-score for each gene. This combined statistic was a directional representation of change between allograft dysfunction and stably functioning grafts across datasets. To identify sets of genes that were changing, Wilcoxon rank-sum tests were performed on the combined *P* values that were determined for each gene within our gene set analysis, returning a significance value for KEGG^66^ and Reactome^67^ pathways that were enriched in the dysfunction of interest. Where we wanted to infer a directional change, a gene set enrichment analysis (GSEA) was performed on those ranked lists of genes, using the clusterProfiler package in R as well as the KEGG and Reactome databases.

#### Determining cell-type-specific gene set

To establish which cell type expressed genes found in our meta-analysis, we made use of the Cepo package, which identifies cell identity genes for cell types^68^. Treating each sample as a distinct dataset, genes were ranked based on their relative importance to a particular cell type using Cepo statistics. These statistics were then clustered using the Pearson correlation coefficient to evaluate the specificity of the cell type signal within the allograft. Subsequently, using the Cepo statistics, we used a Wilcoxon rank-sum test to compute the enrichment of the genes identified in our meta-analysis across different cell types. We compared the results across sample conditions, namely acute rejection and stably functioning grafts.

### TOP framework

To address the challenge of building a robust and predictive model across different datasets and platforms, we developed the TOP framework. TOP is a modeling approach that constructs reference-free biomarkers, which are required to yield consistent predictions across data from different platforms in the absence of internal normalization standards^30–32^. An extension of our previous model, CPOP^26^, this transfer learning framework is designed to construct prediction models that (1) are self-normalizing and so can be applied across platforms without relying on traditional batch correction methods and (2) ensure that all organs contribute equally to the model construction. This predictive framework is available on the Bioconductor Project: https://bioconductor.org/packages/release/bioc/html/TOP.html.

In brief, the TOP framework starts by defining a set of features that consistently change across all the datasets used to train the model. The top 50 genes that are most differentially expressed are selected for subsequent analysis and used to create a log-ratio matrix (Supplementary Methods). To identify important features for the model, the fold change for each ratio in relation to a binary outcome was calculated in all the individual datasets used for training. The fold changes were then combined using a weighted mean scaled by their variance, to ensure that selected features were changing in all organs (Supplementary Methods). The scores were then used to weight features in a lasso regression model. The lasso was chosen for its ability to shrink coefficients to zero, producing sparse models.

#### Motivation for TOP framework

With the goal of creating a pan-organ model from a diverse range of publicly available datasets, we developed a method of modeling omics data across organs. Traditional approaches to build models across datasets fall under the umbrella of batch correction. However, within our training datasets, both batch and organ are perfectly confounded. Hence, traditional batch correction methods would not be applicable.

Our objective was to create a comprehensive pan-organ model capable of classifying allograft rejection instances across all transplanted organs. Considering that our data collection encompassed heart, lung, liver and kidney transplant recipients, it was crucial for the model to achieve equilibrium among all organs to be considered genuinely pan-organ. Consequently, we designed the TOP framework. This framework seeks to accomplish three primary goals:

1. Maintain balance among all organs in the framework, preventing overemphasis on the more abundant kidney transplant datasets while disregarding smaller ones.
2. Assign equal weight to each study in our atlas, avoiding disproportionate learning from datasets with particularly large sample sizes.
3. Yield transparent and interpretable coefficients, thereby facilitating a smooth transition to the identification of potential biomarkers.

#### TOP methodology

Suppose we want to fit a model across datasets, then ${X}_{\rm{i}}={X}_{1},{X}_{2},{X}_{3},\ldots {X}_{\rm{k}}$, where ${X}_{\rm{i}}\in {{\mathbb{R}}}^{\rm{n\times p}}$. First, we define a set of features ${S}^{\rm{(i)}}$ to be the intersection of features in ${X}_{\rm{i}}$. Then, we can redefine ${X}_{\rm{i}}$ as ${X}_{\rm{i(s)}}$. Let a vector $y\in {{\mathbb{R}}}^{\rm{n}}$ represent a patient’s clinical outcome (for example, biopsy-proven rejection). We, therefore, can define a moderated test statistic for each feature within ${X}_{i\left(S\right)}$ using the limma package. The moderated test statistic is assumed to follow a Gaussian distribution and so is converted to a z-score and subsequent *P* value^28,29^. Stouffer’s method of combining *P* values is then used to determine features that are important across all datasets. These features are ranked according to combined *P* value, and, by default, the top 50 are included for subsequent analysis. Consequently, we subset ${X}_{\rm{i}}$ once more, incorporating only the top 50 features in each dataset, resulting in an updated matrix ${X}_{\rm{i}}({\rm{top}}50)$.

#### Creating the log-ratio matrix

The ‘log-ratio matrix’ was first proposed by Wang et al.^26^. In brief, a matrix $Z$ of dimension ${{\mathbb{R}}}^{\rm{k\times q}}$ where and each column of $Z$ represents the pairwise difference between two log-transformed columns in ${X}_{\rm{i}\left({top}50\right)}\in {{\mathbb{R}}}^{\rm{k\times p}}$. Specifically, each column of $Z$ consists of all log-ratio features for $1\le l < m\le p$, signifying that each column in the $Z$ matrix is the log-ratio of the expression values of two features. For the given log-ratio matrix $Z\in {{\mathbb{R}}}^{\rm{k\times q}}$, we denote each row of the matrix as ${Z}_{\rm{i}}$ for sample $i=1,\ldots ,k$.

Following the methodology outlined above, we proceed to calculate the log fold change for each feature in relation to the binary outcome $y\in {{\mathbb{R}}}^{k}$ within each $Z$ matrix. Keep in mind that there may be up to ${Z}_{\rm{k}}$ matrices, with *k* signifying the total number of datasets. For every matrix ${Z}_{\rm{i}}$, where $i=1,\ldots ,k$, we determine the log fold change by contrasting the expression values of each feature between the two groups delineated by the binary outcome $y$.

#### Calculating feature weights

To address the concern that larger datasets might overpower the signal from smaller datasets, we can calculate weights for each dataset $i=\{\mathrm{1,2},\ldots ,k\}$ based on their respective sample sizes. This approach ensures that datasets with a larger number of samples do not disproportionately influence the overall analysis. To calculate the weighted mean (${\mu }_{\rm{w}}$) of the log fold changes, we first assign a weight (${w}_{i}$) to each dataset, corresponding to the inverse of its sample size. The weighted mean (${\mu }_{\rm{w}}$) for each dataset *i* is then computed as follows:

$${\mu }_{\rm{w}}=\frac{\mathop{\sum }\nolimits_{\rm{i=1}}^{\rm{k}}{w}_{\rm{i}}* {x}_{\rm{i}}}{\mathop{\sum }\nolimits_{\rm{i=1}}^{k}{w}_{\rm{i}}}$$

where ${x}_{\rm{i}}$ denotes the log fold change for dataset *i*, and $k$ represents the total number of datasets.

Similarly, to determine the weighted variance (${\sigma }_{\rm{w}}^{2}$), we use the formula:

$${\sigma }_{\rm{w}}^{2}=\frac{\mathop{\sum }\nolimits_{\rm{i=1}}^{k}\left({{w}_{\rm{i}}* ({x}_{\rm{i}}-{\mu }_{\rm{w}})}^{2}\right)}{\mathop{\sum }\nolimits_{\rm{i=1}}^{k}{w}_{\rm{i}}}$$

By employing these weighted calculations, we ensure that the analysis accommodates the varying sizes and characteristics of the datasets from different organ transplant cohorts, ultimately providing a more robust and reliable assessment of the log fold changes across all datasets.

Next, we compute a test statistic for each ratio, which is obtained by dividing its mean change by its variance. Additionally, to ensure stability and mitigate the impact of extreme values, we introduce a fudge factor in the denominator. We denote it as ${Q}_{0.9}({\sigma }_{\rm{w}}^{2})$, where ${Q}_{0.9}$ denotes the 90th quantile function applied to the weighted variances (${\sigma }_{\rm{w}}^{2}$). Consequently, the test statistic ${T}_{\rm{j}}$ for each ratio $j$ can be calculated using the following formula:

$${T}_{\rm{j}}=\frac{{\mu }_{\rm{wj}}}{{\sigma }_{\rm{wj}}^{2}+{Q}_{0.9}({\sigma }_{\rm{w}}^{2})}$$

where ${\mu }_{\rm{wj}}$ represents the weighted mean of the log fold change for ratio $j=\{\mathrm{1,2},\ldots ,q\}$. ${\sigma }_{\rm{wj}}^{2}$ denotes the corresponding weighted variance, and $q$ signifies the total number of ratios.

By incorporating the fudge factor, we account for potential outliers and ensure that the test statistic remains robust in the presence of features with small variances across datasets. This approach enhances the reliability of the analysis, contributing to a more accurate assessment of the relationships between features and clinical outcomes.

Finally, to smooth the effects of weighting features, we take the square root of each test statistic ${T}_{\rm{j}}$, giving us a transformed weight of each feature ${w}_{\rm{f}}$.

#### Calculating organ (sample) weights

To account for the balance among the four organ transplants discussed earlier, we proceed to compute a weight for each observation. We can define the weight (${w}_{\rm{i}}$) for each organ $i$ as proportional to the inverse of its number of datasets (${n}_{\rm{i}}$): ${w}_{\rm{i}}=\frac{1}{\sqrt[4]{{n}_{\rm{i}}}}$ for $i=1,\ldots ,k$. This weighting strategy, combined with additional smoothing, ensures that no single organ transplant type disproportionately influences the results while accounting for the inherent right skew in our training set.

#### Building the lasso model

Both the feature and sample size weights are incorporated into a weighted lasso regression model. The lasso was chosen for the ability to force features out of the model, providing concise estimates of feature importance. The lasso is built by first concatenating the log-ratio matrices ${Z}_{\rm{i}}$ for $i=\{\mathrm{1,2},\ldots ,k\}$.

$$\hat{\beta }(\,y,Z\left|\right.{w}_{\rm{f}},{w}_{\rm{s}},\lambda)=\mathop{\min }\limits_{\beta \in {{\mathbb{R}}}^{q}}\mathop{\sum }\limits_{\rm{i=1}}^{k}{{w}_{{si}}\left(\,{y}_{\rm{i}}-{\beta }_{0}-\mathop{\sum }\limits_{\rm{j=1}}^{q}{Z}_{\rm{i}}^{\text{T}}{w}_{\rm{fj}}{\beta }_{\rm{j}}\right)}^{2}+\lambda \mathop{\sum }\limits_{\rm{j=1}}^{q}\left|{\beta }_{\rm{j}}\right|$$

#### Building the pan-organ transfer learning model

To create a robust pan-organ model capable of accurately classifying instances of allograft rejection across all transplanted organs, we amalgamated rejection phenotypes across organs. This addressed the challenges posed by the lack of uniform definitions of organs and the evolution of histopathological guidelines over time. Specifically, we consolidated T-cell-mediated rejection (TCMR), antibody-mediated rejection (ABMR) and mixed phenotypes under one comprehensive definition of rejection. With this composite outcome, a transfer learning model was constructed using the TOP framework. This model was trained using datasets from kidney, lung, liver and heart transplantation. The TOP framework was used to balance feature selection and sample weighting to ensure that each organ was contributing equally to the model, despite disparities in dataset sizes.

#### Transfer learning model evaluation

Both the pan-organ and organ-specific models were evaluated using an LOOCV strategy, whereby models were systemically trained on all available datasets, excluding a testing dataset. AUC was used to quantify model performance.

### AUSCAD

#### Study overview

The AUSCAD is a single-center, prospectively recruited observational cohort study at Westmead Hospital in Australia. Consent was obtained before transplantation with procedures approved by the Western Sydney Local Health District Human Research Ethics Committee (HREC/12/WMEAD/190). Demographic and clinical data, as well as blood and kidney biopsies, were collected at implantation and at 3 months after transplantation. No statistical methods were used to pre-determine sample sizes, but our sample sizes are similar to those reported in previous publications available in our PROMAD atlas (Supplementary Table 1).

#### Sample collection and histopathological evaluation

Two biopsy cores were taken at each protocol or for-cause biopsy, with one used for histology and the other for bulk RNA-seq (described below). Biopsy cores reserved for histology underwent hematoxylin and eosin, periodic acid–Schiff, Masson’s trichrome and C4d staining at the Institute of Clinical Pathology and Medical Research (Westmead Hospital) before evaluation by a single histopathologist, using the Banff 2019 schema^69^.

#### RNA isolation and sequencing

Kidney biopsy specimens were left in RNAlater (Sigma-Aldrich) overnight at 4 °C before removal and storage at −80 °C until RNA extraction. Specimens were chemically and physically lysed by using 2-mercaptoethanol (Sigma-Aldrich) and TissueLyser II (Qiagen), followed by RNA extraction using AllPrep DNA/RNA/microRNA and MiniElute clean-up kits (Qiagen). Peripheral blood was collected into PAXgene Blood RNA tubes (Qiagen), left at room temperature for 5 h and stored at −80 °C until RNA extraction. RNA was extracted by using a PAXgene Blood miRNA Kit (Qiagen). All RNA samples were frozen and stored at −80 °C and then sent in bulk to the Australian Genome Research Facility. Sample quality control and library preparation were performed in-house, and the resultant libraries were sequenced using the NovaSeq 6000 platform (Illumina) with 100-bp, paired-end read length.

#### Downstream analysis and normalization

Raw FASTQ files were first trimmed and aligned using the GRCh37-hg19 reference genome. The resulting data were then organized into a gene counts matrix for each sample. The bulk RNA-seq data underwent initial filtering to remove reads too low for further analysis^70^. This was followed by normalization using the TMM method^25^.

### External validation

#### Liquid biopsy validation

We evaluated our pan-organ liquid biopsy model on prospectively collected blood samples. Outcomes associated with each blood sample were assessed using corresponding biopsy scores. The AUSCAD validation set also used a composite definition of rejection (using Banff 2019 criteria^69^).

#### Data-derived gene set validation

To evaluate the clinical relevance of our data-derived gene set for global allograft dysfunction and the BHOT panel, we conducted a validation study using the AUSCAD cohort. We constructed three pan-organ models using our TOP method to predict DGF (defined as requiring dialysis within 1 week of transplantation), allograft rejection (a composite value as described above) and fibrosis, based on biopsy data from 7, 54 and 14 PROMAD atlas datasets, respectively. Instead of performing feature selection, we incorporated features from our data-derived gene set (*n* = 500) or the BHOT panel (*n* = 770) in model development. Our DGF model was assessed in 136 AUSCAD pre-implantation biopsies. For allograft rejection, 121 biopsies taken 3 months after transplantation were analyzed. The fibrosis model was tested on 86 biopsies with interstitial fibrosis and tubular atrophy (IFTA) scores over 10% and no concurrent rejection, as determined by a single pathologist. AUC was used as an evaluation metric to compare model performance.

### Reporting summary

Further information on research design is available in the Nature Portfolio Reporting Summary linked to this article.

## Online content

Any methods, additional references, Nature Portfolio reporting summaries, source data, extended data, supplementary information, acknowledgements, peer review information; details of author contributions and competing interests; and statements of data and code availability are available at 10.1038/s41591-024-03030-6.

## Supplementary information

Supplementary Information Supplementary Figs. 1 and 2.

Reporting Summary

Supplementary Tables 1 and 2.

## Data Availability

The data used in this manuscript are publicly available on the Gene Expression Omnibus (https://www.ncbi.nlm.nih.gov/geo/) and ArrayExpress (https://www.ebi.ac.uk/arrayexpress/). The accession codes for each individual study are supplied in Supplementary Table 1. Furthermore, all processed data used in this study are available for download at https://shiny.maths.usyd.edu.au/PROMAD/. AUSCAD RNA-seq data, derived from peripheral blood samples collected 3 months after transplant, are publicly accessible in the Gene Expression Omnibus database (accession code GSE248752). RNA-seq data from biopsy samples taken before graft re-perfusion are available under accession code GSE261240, and those from biopsy samples obtained 3 months after transplant can be found under GSE261892.

## Code availability

The code used to analyze all datasets presented in this manuscript and in the figures is available on GitHub (https://github.com/Harry25R/POAD_Figures).
