# Supplementary material for: Decoding the hallmarks of allograft dysfunction with a comprehensive pan-organ transcriptomic atlas
Source: Nat Med. 2024 Jun 18;30(12):3748–57. doi: 10.1038/s41591-024-03030-6 (PMC11645273; doi:10.1038/s41591-024-03030-6)
Supplement: Supplementary file 1 — Supplementary Figs. 1 and 2. [file 41591_2024_3030_MOESM1_ESM.pdf]

nature medicine

Supplementary information https://doi.org/10.1038/s41591-024-03030-6

Decoding the hallmarks of allograft dysfunction with a comprehensive pan-organ transcriptomic atlas

In the format provided by the authors and unedited

Supplementary Material

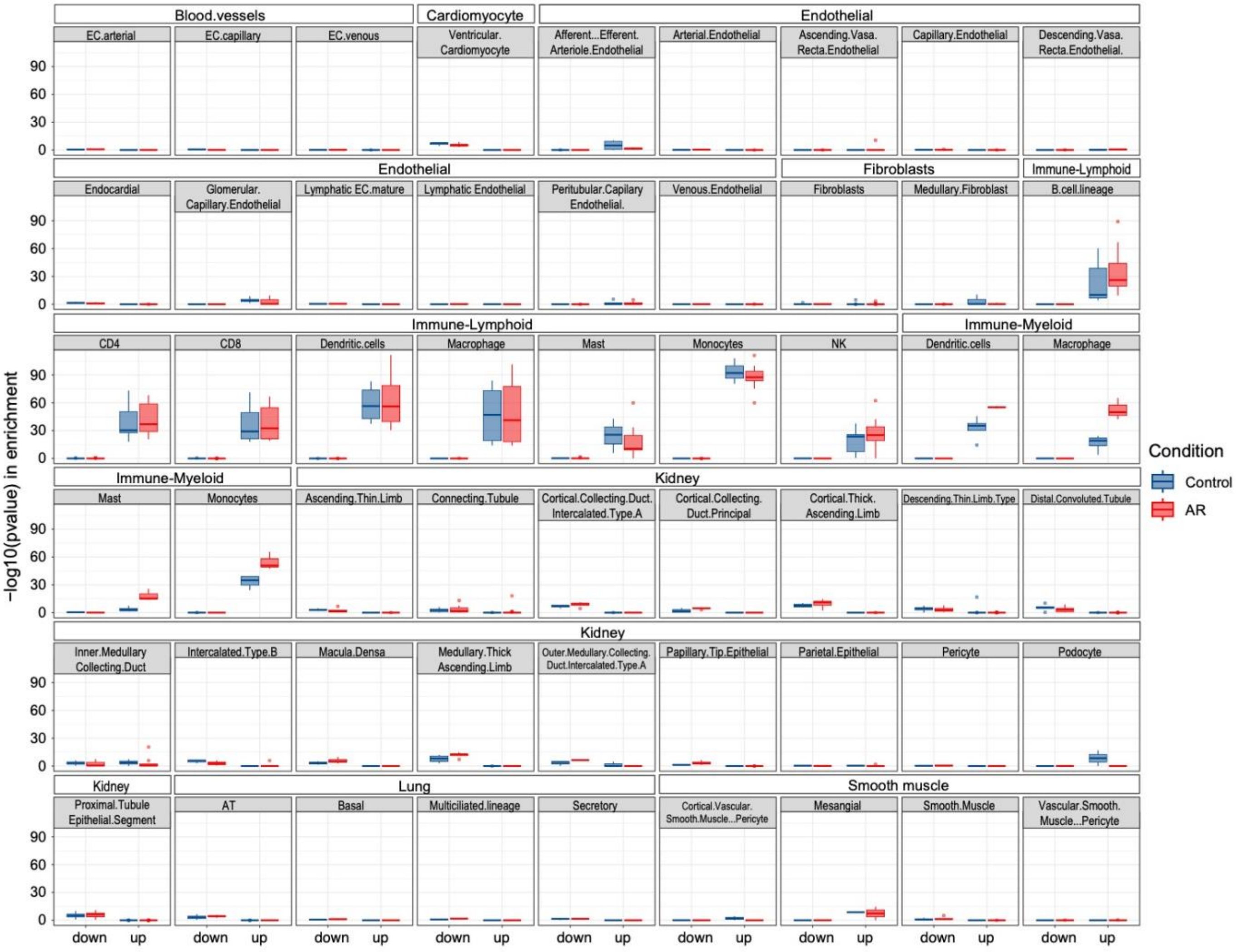

Supplementary Figure 1: Preferential enrichment of the signature genes in minor cell types from allograft rejection.

Boxplot showing the results of gene set enrichment analysis (GSEA) on the cell-type-specific identity statistics. The degree of enrichment is indicated by the -log10 P-Value (y-axis) of enrichment of the signature genes. P-Values were adjusted for multiple comparisons using the benjamini-hochberg correction. The results are separated by Control (blue) or allograft rejection (AR; red). (n = 6 and n = 16 biologically independent control and allograft rejection samples were used respectively). Box plots show Q1, median and Q3, and the lower and upper whiskers show Q1 – 1.5 × IQR and Q3 + 1.5 × IQR, respectively. Grey header bars denote the tissue compartment of origin and cell type.

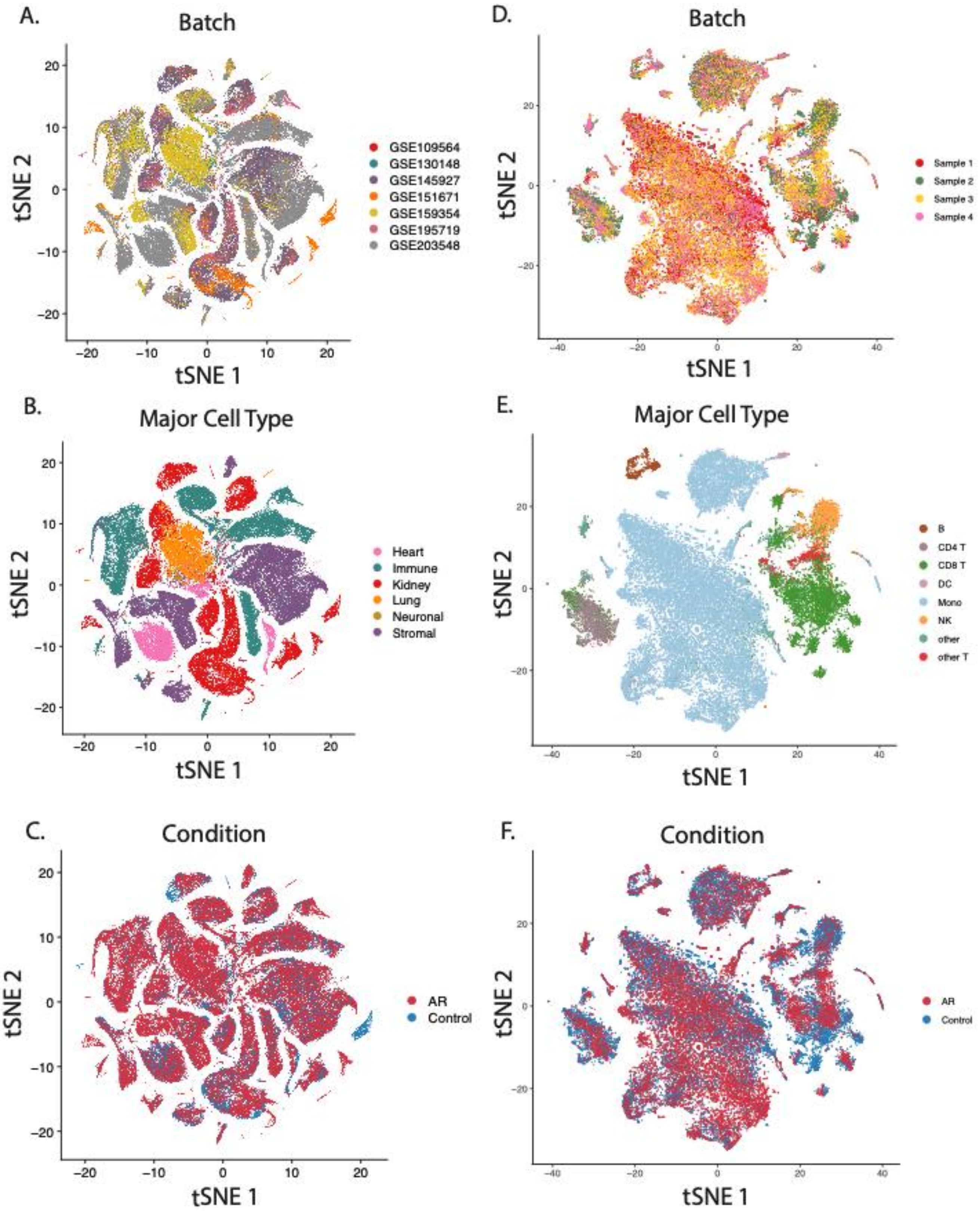
A.
Batch
tSNE 2
tSNE 1
GSE109564
GSE130148
GSE145927
GSE151671
GSE159354
GSE195719
GSE203548
B.
Major Cell Type
Heart
Immune
Kidney
Lung
Neuronal
Stromal
C.
Condition
AR
Control
D.
Batch
Sample 1
Sample 2
Sample 3
Sample 4
E.
Major Cell Type
B
CD4 T
CD8 T
DC
Mono
NK
other
other T
F.
Condition
AR
Control

Supplementary Figure 2: Dimension reduction plots of merged single cell RNAseq data from the PROMAD atlas. A-C. Single Cell RNAseq from Allograft Biopsy Samples A. tSNE projection of the cells of the pan-organ allograft dysfunction atlas. Single cells are coloured by their dataset of origin. **B.** tSNE projection of the cells of the pan-organ allograft dysfunction atlas. Single cells are coloured by tissue origin. **C.** Visualisation of the overall signature score of markers denoting allograft dysfunction. Individual cells are coloured by the signature score, where a darker colour denotes higher expression of signature genes. The overall score is determined by averaging the gene expression of 694 markers. **D-F. Single Cell RNAseq from Peripheral Blood Samples from Transplant Recipients. D.** tSNE project of cells from the pan-organ allograft dysfunction atlas. Single cells are coloured by their sample of origin. **E.** tSNE projection of cells from the pan-organ allograft dysfunction atlas. Single cells are coloured by the clinical condition designated by histopathology scoring of biopsy samples. **F.** tSNE projection of the cells of the pan-organ allograft dysfunction atlas. Single cells are coloured by minor cell types, as defined by our PROMAD atlas.
