# Supplementary material for: Decoding the hallmarks of allograft dysfunction with a comprehensive pan-organ transcriptomic atlas
Source: Nat Med. 2024 Jun 18;30(12):3748–57. doi: 10.1038/s41591-024-03030-6 (PMC11645273; doi:10.1038/s41591-024-03030-6)
Supplement: Supplementary file 2 — Reporting Summary [file 41591_2024_3030_MOESM2_ESM.pdf]

nature portfolio

Corresponding author(s): Dr. Ellis Patrick
Last updated by author(s): Apr 5, 2024

Reporting Summary

Nature Portfolio wishes to improve the reproducibility of the work that we publish. This form provides structure for consistency and transparency in reporting. For further information on Nature Portfolio policies, see our Editorial Policies and the Editorial Policy Checklist.

Statistics

For all statistical analyses, confirm that the following items are present in the figure legend, table legend, main text, or Methods section.

n/a	Confirmed	
☐	☒	The exact sample size (*n*) for each experimental group/condition, given as a discrete number and unit of measurement
☐	☒	A statement on whether measurements were taken from distinct samples or whether the same sample was measured repeatedly
☐	☒	The statistical test(s) used AND whether they are one- or two-sided<br>*Only common tests should be described solely by name; describe more complex techniques in the Methods section.*
☐	☒	A description of all covariates tested
☐	☒	A description of any assumptions or corrections, such as tests of normality and adjustment for multiple comparisons
☐	☒	A full description of the statistical parameters including central tendency (e.g. means) or other basic estimates (e.g. regression coefficient) AND variation (e.g. standard deviation) or associated estimates of uncertainty (e.g. confidence intervals)
☐	☒	For null hypothesis testing, the test statistic (e.g. *F*, *t*, *r*) with confidence intervals, effect sizes, degrees of freedom and *P* value noted<br>*Give P values as exact values whenever suitable.*
☒	☐	For Bayesian analysis, information on the choice of priors and Markov chain Monte Carlo settings
☒	☐	For hierarchical and complex designs, identification of the appropriate level for tests and full reporting of outcomes
☒	☐	Estimates of effect sizes (e.g. Cohen's *d*, Pearson's *r*), indicating how they were calculated

Our web collection on statistics for biologists contains articles on many of the points above.

Software and code

Policy information about availability of computer code

Data collection	No software was used for the collection of data. All data was downloaded from the GEO portal using the GEOquery package in R, version 2.64.2
Data analysis	Data was analysed using R version 4.2.0. limma version 3.54.0, directPA version 1.5, clusterProfiler version 4.4.4, Cepo version 1.2, TOP version 0.99.0.

For manuscripts utilizing custom algorithms or software that are central to the research but not yet described in published literature, software must be made available to editors and reviewers. We strongly encourage code deposition in a community repository (e.g. GitHub). See the Nature Portfolio guidelines for submitting code & software for further information.

Data

Policy information about availability of data

All manuscripts must include a data availability statement. This statement should provide the following information, where applicable:

- Accession codes, unique identifiers, or web links for publicly available datasets
- A description of any restrictions on data availability
- For clinical datasets or third party data, please ensure that the statement adheres to our policy

The data used in this manuscript is publicly available on the Gene Expression Omnibus (https://www.ncbi.nlm.nih.gov/geo/) and ArrayExpress (https://www.ebi.ac.uk/arrayexpress/). The accession codes for each individual study are supplied in Supplementary Table 1. Further, all processed data used in this study is

available for download at https://shiny.maths.usyd.edu.au/PROMAD/.
AUSCAD RNA sequencing data, derived from peripheral blood samples, collected 3-months post-transplant, is now publicly accessible in the GEO database (accession number GSE248752). RNA sequencing data from biopsy samples taken prior to graft reperfusion are available under the accession code GSE261240, and those from biopsy samples obtained three months post-transplant can be found under GSE261892.

Research involving human participants, their data, or biological material

Policy information about studies with human participants or human data. See also policy information about sex, gender (identity/presentation), and sexual orientation and race, ethnicity and racism.

Reporting on sex and gender	Our study collected the biological sex of the recipient and has been reported in the associated data tables. Gender information was not collected as part of the study.
Reporting on race, ethnicity, or other socially relevant groupings	Our study did not accurately collected the race or ethnicity of the recipient. Moreover, we are unable to provide specific donor characteristics due to patient privacy.
Population characteristics	We are unable to provide specific donor characteristics due to patient privacy. However, we have included relevant recipient characteristics that are important to our study. Specifically, we have included comparisons of clinical data between rejection and control groups including biological sex.
Recruitment	Patients were prospectively recruited from Westmead Hospital in Sydney, Australia. All patients were consented by the on-call physician at the time of the transplant for their inclusion in the study. There was no additional criteria for recruitment. Patients who were recruited received that same standard of care as those that did not as this was an observational study.
Ethics oversight	Western Sydney Local 221 Health District Human Research Ethics Committee (HREC/12/WMEAD/190)

Note that full information on the approval of the study protocol must also be provided in the manuscript.

Field-specific reporting

Please select the one below that is the best fit for your research. If you are not sure, read the appropriate sections before making your selection.

☒ Life sciences ☐ Behavioural & social sciences ☐ Ecological, evolutionary & environmental sciences

For a reference copy of the document with all sections, see nature.com/documents/nr-reporting-summary-flat.pdf

Life sciences study design

All studies must disclose on these points even when the disclosure is negative.

Sample size	A sample size ranged due to patient drop out and sample collection. Initially we had 136 patients that had pre-implant biopsies collected. 121 of those patients were followed up 3-months post transplantation. Blood was collected and sequenced in 70 of those 121 patients, as blood collection was not the primary aim of the AUSCAD study. Our observational study is still undergoing recruitment however we have already achieved sample size similar to other such studies in our PROMAD atlas (Supplementary Table 1)
Data exclusions	There was no exclusion criteria after samples had been collected in our study.
Replication	All code required to process the raw data and reproduce associated figures has been provided in the manuscript.
Randomization	Our study measured the occurrence of a particular pathology in organ transplant patients. All patients recieved the same standard of care and samples were collected at the same time. Therefore, randomization was not relevant to our study.
Blinding	During data analysis, all patient data was deidentified to authors. When assessing model performance, predictions were married with the outcome as defined by a single pathologist, and evaluation metrics calculated.

Reporting for specific materials, systems and methods

We require information from authors about some types of materials, experimental systems and methods used in many studies. Here, indicate whether each material, system or method listed is relevant to your study. If you are not sure if a list item applies to your research, read the appropriate section before selecting a response.

Materials & experimental systems

n/a	Involved in the study
☒	☐ Antibodies
☒	☐ Eukaryotic cell lines
☒	☐ Palaeontology and archaeology
☒	☐ Animals and other organisms
☒	☐ Clinical data
☒	☐ Dual use research of concern
☒	☐ Plants

Methods

n/a	Involved in the study
☒	☐ ChIP-seq
☒	☐ Flow cytometry
☒	☐ MRI-based neuroimaging

Plants

Seed stocks

Report on the source of all seed stocks or other plant material used. If applicable, state the seed stock centre and catalogue number. If plant specimens were collected from the field, describe the collection location, date and sampling procedures.

Novel plant genotypes

Describe the methods by which all novel plant genotypes were produced. This includes those generated by transgenic approaches, gene editing, chemical/radiation-based mutagenesis and hybridization. For transgenic lines, describe the transformation method, the number of independent lines analyzed and the generation upon which experiments were performed. For gene-edited lines, describe the editor used, the endogenous sequence targeted for editing, the targeting guide RNA sequence (if applicable) and how the editor was applied.

Authentication

Describe any authentication procedures for each seed stock used or novel genotype generated. Describe any experiments used to assess the effect of a mutation and, where applicable, how potential secondary effects (e.g. second site T-DNA insertions, mosiacism, off-target gene editing) were examined.
